# Monitoring Parkinson’s disease symptoms during daily life: a feasibility study

**DOI:** 10.1038/s41531-019-0093-5

**Published:** 2019-09-30

**Authors:** Margot Heijmans, Jeroen G. V. Habets, Christian Herff, Jos Aarts, An Stevens, Mark L. Kuijf, Pieter L. Kubben

**Affiliations:** 1 School of Mental Health and Neuroscience, Maastricht University, Maastricht, Netherlands; 2 Instrument Development Engineering & Evaluation, Maastricht University Medical Center, Maastricht, Netherlands; 3 Department of Neurology, Maastricht University Medical Center, Maastricht, Netherlands; 4 Department of Neurosurgery, Maastricht University Medical Center, Maastricht, Netherlands

**Keywords:** Parkinson's disease, Parkinson's disease, Parkinson's disease

## Abstract

Parkinson’s disease symptoms are most often charted using the MDS-UPDRS. Limitations of this approach include the subjective character of the assessments and a discrepant performance in the clinic compared to the home situation. Continuous monitoring using wearable devices is believed to eventually replace this golden standard, but measurements often lack a parallel ground truth or are only tested in lab settings. To overcome these limitations, this study explores the feasibility of a newly developed Parkinson’s disease monitoring system, which aims to measure Parkinson’s disease symptoms during daily life by combining wearable sensors with an experience sampling method application. Twenty patients with idiopathic Parkinson’s disease participated in this study. During a period of two consecutive weeks, participants had to wear three wearable sensors and had to complete questionnaires at seven semi-random moments per day on their mobile phone. Wearable sensors collected objective movement data, and the questionnaires containing questions about amongst others Parkinson’s disease symptoms served as parallel ground truth. Results showed that participants wore the wearable sensors during 94% of the instructed timeframe and even beyond. Furthermore, questionnaire completion rates were high (79,1%) and participants evaluated the monitoring system positively. A preliminary analysis showed that sensor data could reliably predict subjectively reported OFF moments. These results show that our Parkinson’s disease monitoring system is a feasible method to use in a diverse Parkinson’s disease population for at least a period of two weeks. For longer use, the monitoring system may be too intense and wearing comfort needs to be optimized.

## Introduction

Parkinson’s disease (PD) is a neurodegenerative disorder characterized by motor symptoms like tremor, rigidity and bradykinesia, which can fluctuate during the day. Nowadays, symptoms and disease course are charted by a clinician and are measured most often using the MDS-UPDRS for research purposes.^1^ This is however associated with limitations: (1) symptoms and disease course are assessed with low frequency and therefore only permit a snapshot of the clinical situation and may include recall bias; (2) scores given by the clinician have a subjective character; (3) patients might put themselves in a better light during in-clinical assessments compared to when they are at home. In order to get more frequent and more objective ratings of symptoms and disease course, continuous monitoring systems are essential. Such systems are believed to represent the clinical symptoms in the daily life of patients more reliably and could eventually even be used as input signal for adaptive, responsive, or closed-loop deep brain stimulation (DBS).^2^

Wearable sensors are increasingly used to detect PD symptoms due to technological innovations, resulting in small, low cost, power efficient, and accurate sensors.^3–5^ Wearable sensors have shown promise in detecting tremor,^6–8^ freezing of gait,^9^ bradykinesia,^10^ and dyskinesia.^10,11^ Recently, various monitoring systems have been developed and tested and have shown promising results.^12–14^ These studies made use of predefined motor tasks in a lab or simulated home setting, which only gives a limited representation of the daily life environment. Furthermore, these regular clinical assessments cannot be performed continuously in a daily life environment.

**Fig. 1:**
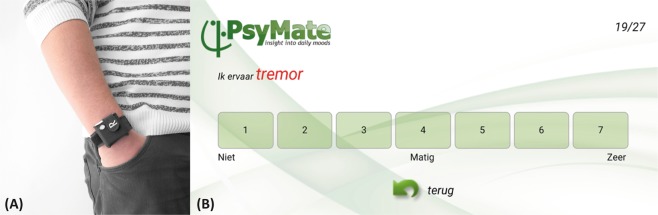
Recording modalities. **a** Wearable sensor attached to the wrist via a wristband. **b** Screenshot of the question ‘Ik ervaar tremor’ (‘I experience tremor’) on a 7 point Likert scale, with a score of 1 indicating not at all and a score of 7 indicating very much

The newly developed PD monitoring system presented in this paper combines wearable sensors measuring acceleration and rotational acceleration with an experience sampling method (ESM) application (Fig. 1). ESM is a validated, digital diary method consisting of multiple repeated measurements at semi-random moments in daily life.^15,16^ It is superior compared to standard diary and cross-sectional assessments, because there is no recall bias and data are collected on multiple moments a day.^17^ The designed ESM questionnaires include questions regarding mood states, contextual information and both motor and non-motor PD symptoms. ESM data may serve as a parallel ground truth to the wearable sensor data, making the impossible regular clinical assessments in daily life measurements redundant. The unique system combines objective data (wearable sensors) with subjective data (ESM) in the daily life of the patients.

Although the combination of wearable sensors with ESM or other electronic diary methods was often suggested in PD,^18,19^ and although this combination was used in other populations before,^20–25^ it has never been tested in PD so far. Consequently, this project aims to prove the feasibility of the new monitoring system in PD patients during daily life for two consecutive weeks. In addition, we investigated whether the ESM answers can be employed as a ground truth for the sensor data by performing an OFF moment prediction analysis. Eventually, we would like to further assess whether this system could also be used for closed-loop DBS programming.

## Results

### Wearable sensors

**Fig. 2:**
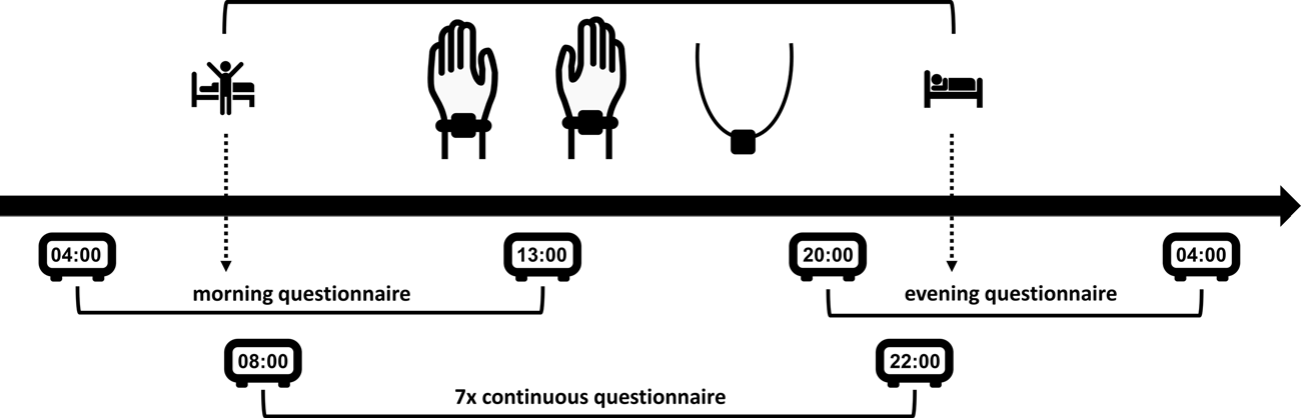
Schematic overview of one test day. Wearable sensors were worn from waking up until going to bed. Questionnaires were available (morning and evening) or showed up (continuous) between the indicated timeframes

On average, the group participants wore the wearable sensors 898 min a day, equalling almost 15 h. The mean time that the wearable sensors were used by the participants was 788 min (94%) within the instructed timeframe of 8.00–22.00 h (Fig. 2). Consequently, the mean non-worn time within the instructed timeframe was 52 min (6%). The mean time that the wearable sensors were used by the participants outside the instructed timeframe was 110 min.

Answers from the evaluation questionnaire showed that most participants found that the wrist (60%) and the chest (80%) sensors were comfortable to wear. However, some participants mentioned that the used accessories remained attached to clothes, or that they caused irritation. Two participants (10%) reported that the wrist sensors impaired their arm movement. According to all participants, the wrist sensors did not impair hand movement nor did the three sensors impair movements in general. None of the participants found the sensors were heavy to wear, had problems with putting the sensors on and off, or had problems with charging the sensors. Almost all participants (95%) wore the sensors in public. Some participants mentioned that they thought it was awkward to get questions about the sensors, and therefore they were glad that it was possible to wear the sensors beneath their clothes. Only 30 and 50% of the participants was willing to wear the wrist and chest sensors on a long-term basis.

### Experience sampling method

**Fig. 3:**
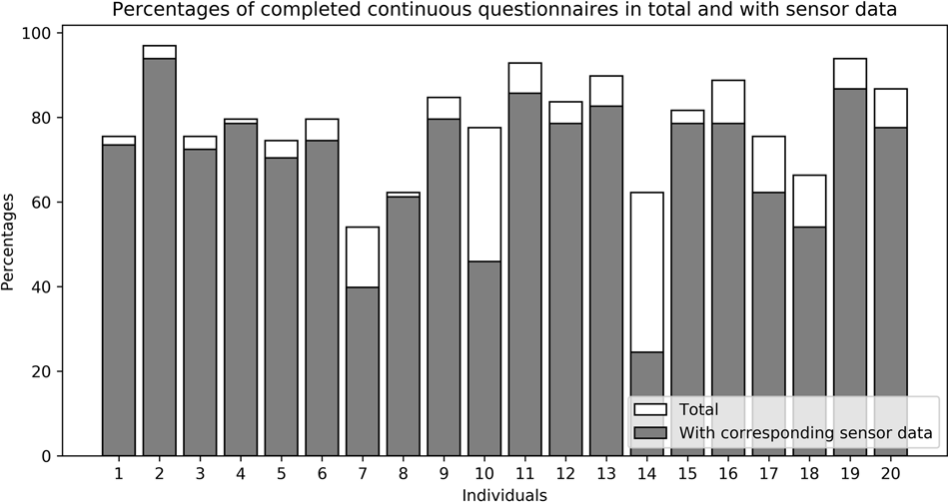
Completion rates. Percentages of total completed continuous questionnaires per participant (white bars) and completed continuous questionnaires with corresponding sensor data (grey bars)

ESM is a validated, digital diary method consisting of multiple repeated measurements at semi-random moments. Participants received a total of 98 continuous questionnaires, 14 morning questionnaires, and 14 evening questionnaires (Fig. 2). Only fully completed questionnaires were considered as completed. On average, 77.5 continuous questionnaires, 13.6 morning questionnaires, and 13.2 evening questionnaires were completed. This resulted in completion rates of 79.1% for the continuous questionnaires, 96.8% for the morning questionnaires and 93.9% for the evening questionnaires. For each completed continuous questionnaire, it was checked whether there was sensor data available from the two wrist sensors and the chest sensor for at least 15 min before the questionnaire was opened by the participant. We hypothesize that this timeframe will best reflect the patients clinical state belonging to the corresponding ESM answer, and therefore this timeframe will be used for further analyses. In total, a mean of 69.0 (89.0%) continuous questionnaires were completed which had corresponding data from all three sensors (Fig. 3). For the majority of participants, only a few continuous questionnaires did not have corresponding sensor data from all three sensors for at least 15 min preceding the questionnaire (Fig. 3). Three participants (7, 10, 14 in Fig. 3) missed sensor data for more than 25% of the completed questionnaires. This was because sensors stopped recording due to full data storage as a result of hidden folders containing the removed data of previous participants. Four other participants had some aborted recordings for still unknown reasons.

Answers from the evaluation questionnaire showed that all participants found the ESM application easy to use and answered the questions independently. The majority of the participants found the ESM questions clear (85%). Some participants mentioned that the line of questioning was inconsistent. For example, a score of 1 was sometimes the most positive option and sometimes the most negative option. Three participants (15%) found it unpleasant to carry their phone with them all day, for example because they could not due to work. Further, 17 participants (85%) did not mind that the continuous questionnaires showed up at random moments during the day, which was supported by the average score of only 1.7 on the question ‘I found this beep disturbing’. This item was scored on a 7 point Likert scale, where 1 corresponded with ‘not disturbing at all’ and 7 corresponded with ‘very disturbing’. Three participants (15%) thought they missed a lot of questionnaires and ten participants (50%) said they would use the ESM application for a longer period than two consecutive weeks.

### The monitoring system in general

All participants reported that the information about the research was clear, and 95% found the aim of the research clear as well. Three participants (15%) considered the study to be incriminating, and two participants (10%) adapted their daily life because of the study. For example, one participant made shorter cycling trips, since this participant was afraid to miss questionnaires.

### Combining ESM and wearable sensor data

**Fig. 4:**
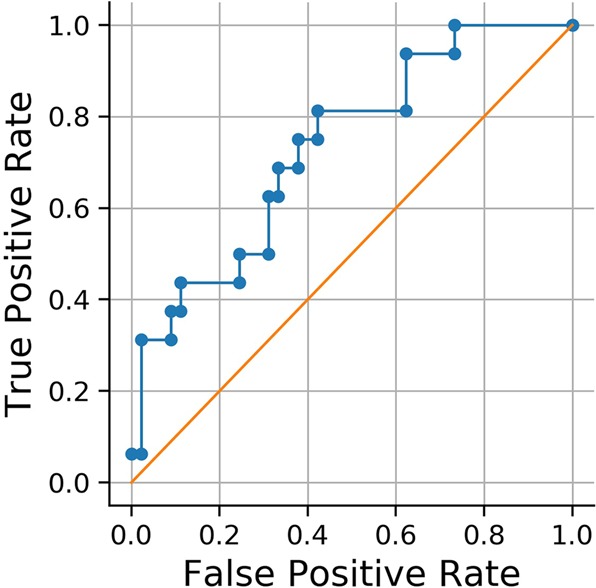
Receiver-Operator-Characteristic curve of detection of OFF moments from sensor data. Aurea under de curve = 0.73

Our pilot investigation on one patient with severe ON/OFF fluctuations yielded a reliable detection of subjectively registered OFF moments based on sensor data using a logistic regression classifier (area under the curve = 0.73). Figure 4 shows true positive and false positive rates for different thresholds (Receiver-Operator-Characteristic). These initial results highlight the feasibility of using wearable sensors to detect symptom severity.

## Discussion

This study showed that combining wearable sensors with ESM is a feasible method for monitoring PD patients in daily life. Results showed that participants wore the wearable sensors almost during the whole instructed timeframe and even beyond, showing that the wearable sensors were not obstructive. Furthermore, ESM completion rates were high and participants evaluated the monitoring system positively. The presented participant characteristics (Table 1) suggest that this monitoring system is feasible for a diverse population of PD patients. For example, age was ranging between 46 and 74 years, disease duration was ranging between 1 and 21 years, and no restrictions were made based on PD phenotype and treatment. There was a relatively high proportion of patients with DBS implants. This selection was most probably due to the population of the academic hospital. We cannot rule out the possibility that PD patients with DBS are more willingly to use technological devices such as this monitoring system.

**Table 1:** Participant characteristics. Data are presented as mean (std)

Male/female, *n*	16/4
Age, years	63.3 (7.4)
Montreal Cognitive Assessment score	27.6 (1.5)
Disease duration, years	8.1 (5.8)
Levodopa equivalent daily dose	770.4 (393.9)
Tremor/akinetic rigid, *n*	9/11
ON/OFF fluctuations yes/no, *n*	12/8
Deep brain stimulation yes/no, *n*	6/14
Deep brain stimulation duration, years	3.3 (1.5)

ESM completion rates were high, with an average of 79%. All participants met the requirement defined in previous work, which demands that at least one third of the continuous questionnaires should be completed to have valid ESM data.^26^ The high completion rate may be due to the extensive briefing during the start session and due to the phone contact moments with the participants on day 2 and 8. Compared to previous work using the same ESM application for 5 days in 5 PD patients, our completion rates were 5% lower.^27^ One might argue that the longer use of the ESM application in this study might explain lower completion rates. This argument is supported by a previous *N* = 1 study, in which the completion rate was 47% in week 1 and dropped to 29% in week 2.^18^ We did however not see any differences between average completion rates per day (Supplementary Fig. 1). Also, there were no differences in completion rates between timeframes (Supplementary Fig. 2). The high completion rates in general are illustrated by the fact that all patients found the application easy to use, and that even half of the patients is willing to use the ESM application for a longer period.

Two participants reported they adapted their daily life during the study period. This was because participants were for example afraid to miss questionnaires. We believe that by using ESM, participants might indeed be more ‘on guard’ and they are required to carry their mobile phone all day. For non-digital questionnaires, there is to our knowledge no study describing whether participants adapted their daily life during the study period. We hypothesize that this will also be the case, since non-digital questionnaires mostly have to be completed at set times. Regarding completion rates it is hard to compare ESM with non-digital diary methods since non-digital diaries can be completed at different moments than instructed.

The evaluation questionnaire outcomes showed that there is room for improvement regarding the used materials of the wearable sensor accessories. The accessories used in this study were developed with the aim that they should be easy to handle for PD patients. This was the case, since none of the participants had trouble with putting the sensors on or off. However, the use of Velcro resulted in the disadvantage that the sensors sometimes remained attached to clothes. Another disadvantage was that the sensors and accessories were not attractive enough to wear. Improvement of the accessories will likely result in a higher amount of patients who find the wearable sensors comfortable to wear and who would wear the wearable sensors on a chronic basis.

**Fig. 5:**
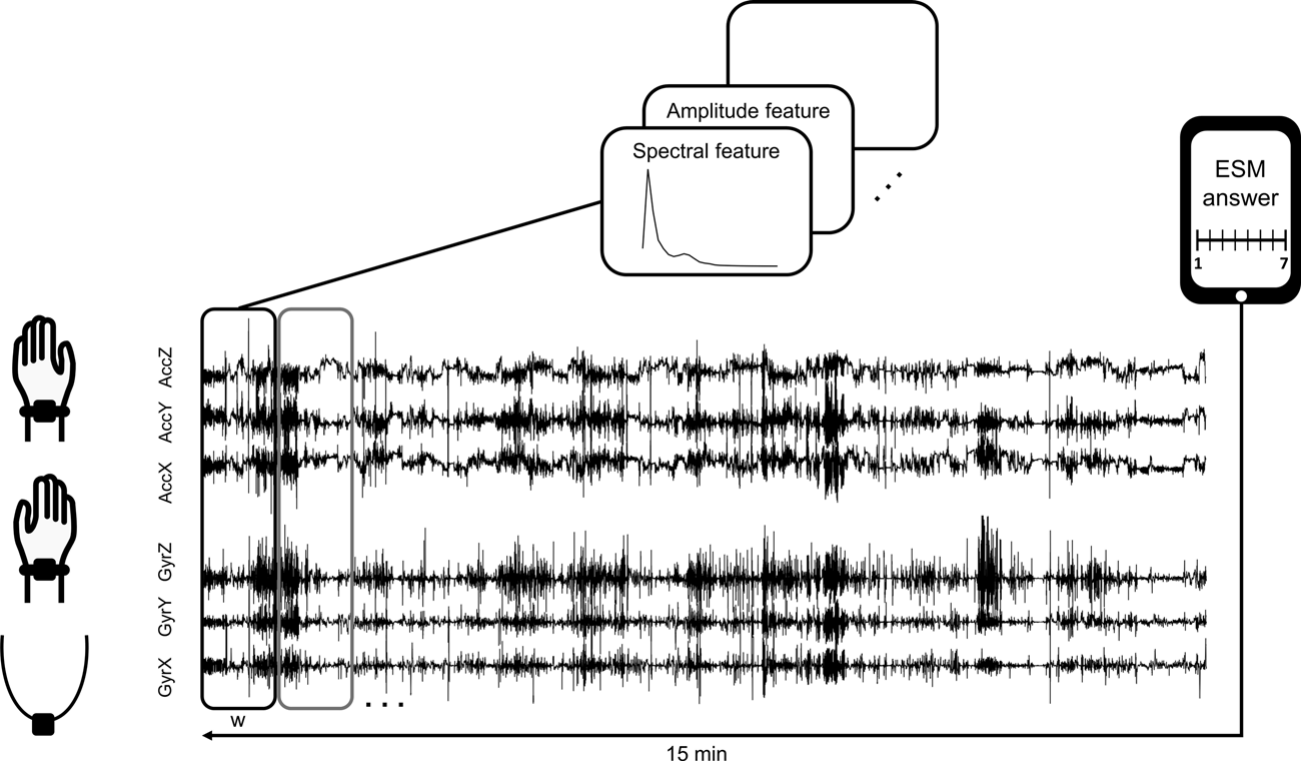
Proposed data processing steps. Fifteen minutes of sensor data prior to a completed questionnaire will be extracted. This timeframe will then be divided into windows of length *w* from which different features in the time and spectral domain will be extracted

The next steps of this study are to validate ESM data, improve PD symptom algorithms for wearable data, correlate wearable data with ESM data, and eventually predict ESM scores based on wearable data. Since the combination of wearable data with ESM data is new in itself, we propose the following data processing steps: for each completed continuous ESM questionnaire, 15 minutes of sensor data prior to this completed questionnaire will be extracted. We hypothesize that this timeframe will best reflect the patients clinical state belonging to the corresponding ESM answer. It should however be tested which timeframe best fits with the questionnaire timestamp. This might be much shorter or longer than the proposed 15 min, or might even be after completion of the questionnaire. Also, it might be necessary to start with an active/inactive classification. On average it took the participants 3 min and 39 s to complete the continuous questionnaire. The start and end time of the completed questionnaires are recorded, so to ensure that the time the questionnaire was completed will not be included in the analysis. The selected timeframe will then be divided into windows of length *w* from which different features in the time and spectral domain will be extracted. Similar to different timeframe lengths, different window lengths for feature extraction will need to be compared as well. The extracted features can then be used for correlation of wearable data with ESM data and eventually for prediction of ESM data. See Fig. 5 for an overview of the proposed data processing pipeline.

One limitation of the used monitoring system is that due to minimizing the number of sensors not all PD symptoms can be measured. For example, tremor in the lower limbs will likely not be measured with this system. Future studies might consider the use of smart insoles as non-obstructive sensor to measure tremor and other symptoms related to the lower limbs.

This study combined wearable sensors and ESM in PD patients. We demonstrated that our newly developed PD monitoring system is feasible and that it can be used for the continuous measurement of PD symptoms during daily life for both monitoring and treatment purposes. Further, it breaks new ground since the system collects objective wearable sensor data as well as subjective ESM data which might be used as a parallel ground truth. Therefore, this monitoring system does not require additional clinical assessments. If correlating wearable and ESM data, and eventually predicting ESM scores based on wearable data succeeds, the monitoring system can be used to monitor the patient during daily life in periods of medication changes, or periods preceding an outpatient clinic visit.

## Methods

### Participants

**Fig. 6:**
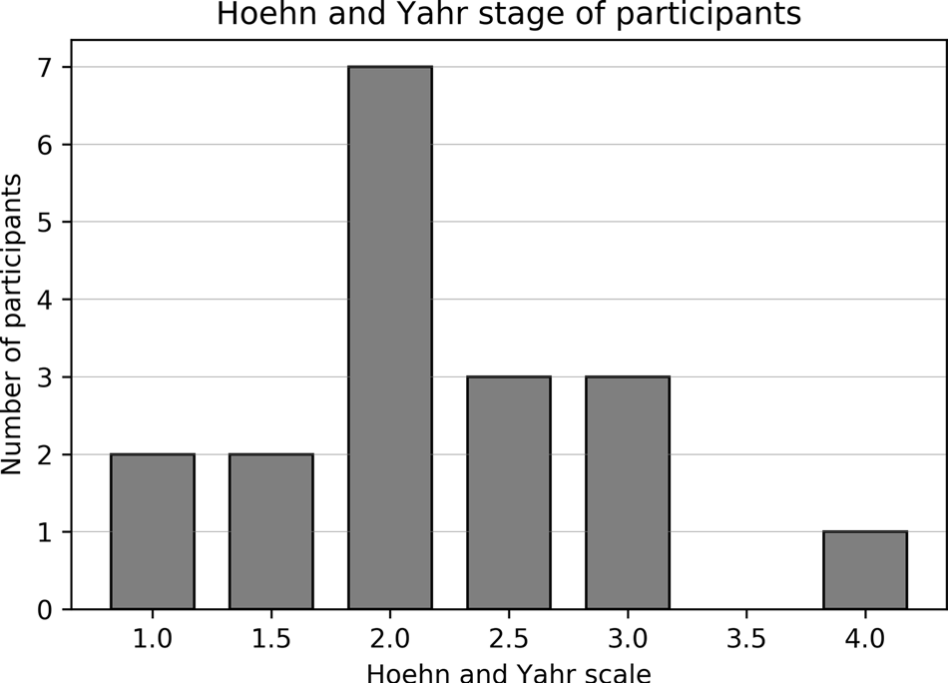
Disease severity of the participants indicated by Hoehn & Yahr scores

This study was approved by the medical ethical committee azM/UM and written informed consent was obtained from all participants. Twenty idiopathic PD patients participated in this study. Recruitment was done through their neurologist or neurosurgeon at the Maastricht University Medical Centre. Disease severity had to be rated as mild to severe (Hoehn and Yahr 1-4). Patients were included if they had an age between 18 and 80 years, if they were in possession of a smartphone (minimal iOS 8 or Android 4), if they could fluently speak and read Dutch, if they were available for two consecutive weeks of representative daily activities (meaning no holidays or planned hospital admission). After written informed consent, participants were tested for cognitive deficits and were excluded if they scored less than 24 points on the Montreal Cognitive Assessment. Participant characteristics are shown in Table 1. Hoehn and Yahr scores are shown in Fig. 6.

### Wearable sensors

We chose to use a new wearable, the MOX5, which was developed by the Instrument Development Engineering & Evaluation department of the Maastricht University. This sensor received CE mark approval and is available for third parties via Maastricht Instruments (Maastricht, The Netherlands). We decided to develop a wearable instead of re-using existing ones because this study acquires access to the raw accelerometer and gyroscope data, and because the on-device data storage removed the need for battery-draining data transfers. Further, this sensor has the possibility to implement symptom algorithms and online data streaming in the future.

The developed sensor contained a 6 DOF sensor, consisting of an accelerometer and gyroscope. The accelerometer covered an amplitude range of ±8 g and the gyroscope covered a range of ±2000 degrees/s. Data were collected with a sampling rate of 200 Hz. During the measurement period, each participant had to wear three wearable sensors; one at each wrist and one at the chest. The wearable sensors were attached to the body via handmade accessories (Fig. 1a). The participants had to wear the wearable sensors during daytime (preferably between 08.00 and 22.00), and had to charge them at night. Wearable sensors were aligned to the ESM questionnaires using time stamps. The participants did not have to interact with the wearable, since the measurement started as soon as the charger was removed and data was later extracted by the research team.

### Experience sampling method

An ESM app, the Psymate, was downloaded and installed on the smartphone of the participants. We developed a specific ESM questionnaire, based on literature discussing relevant symptoms and items for monitoring PD at home^28,29^ and based on patient and clinician interviews about relevant symptoms and items for PD monitoring at home. During the measurement period, participants were asked to complete a morning (five questions) and evening questionnaire (eight questions) which were identical on all days and which were available in the morning and evening (Supplementary Fig. 3). The morning and evening questionnaire were only available during specific timeframes and the participants were asked to complete the questionnaires when they woke up and when they were going to bed. In addition, they received a continuous questionnaire (26 questions) at seven semi-randomized moments during the day (Supplementary Fig. 3). During each 2-hour block between 8.00 and 22.00 h, one continuous questionnaire was sent. See Fig. 2 for an overview of one test day. The continuous questionnaires had to be opened within 15 minutes after the notification alarm and the participants were asked to complete as much questionnaires as possible without adapting their normal daily behaviour. Participants had to rate statement questions on a Likert scale ([1–7], Fig. 1b). Some questions were multiple-choice (Supplementary Fig. 3).

### Combining ESM and wearable sensor data

To investigate whether the ESM answers can be employed as a ground truth for the sensor data, we developed a prediction framework. In this framework, we used features calculated from the sensor data to predict OFF moments experienced by the patient. Based on previous literature^30–32^ the following features were extracted:

1. Logarithmic Signal Energy between 3.5 and 7.5 Hz
2. Root Mean Square of the low-pass filtered (3 Hz) time series
3. Dominant Frequency and dominant energy ratio
4. Amplitude Range of the Raw Time series
5. Maximum Normalized Cross-correlation and corresponding temporal offset between all accelerometer and gyroscope channels.

We evaluated our prediction framework in a 10-fold cross-validation and employed a simple logistic regression classifier to output probabilities for OFF moments. For this pilot study, we only evaluated one patient reporting frequent ON/OFF transitions.

### Outcomes

The feasibility of our PD monitoring system will be expressed in several outcomes. Participants completed an evaluation questionnaire including questions about the use of the wearable sensors and the ESM application and about the study in general. Results of this questionnaire are outcomes for the wearable sensors, ESM and the monitoring system in general.

For the wearable sensors, the outcomes are also the minutes of collected data. This will be divided into three categories: (1) Worn time within instructed timeframe; (2) Non-worn time within instructed timeframe; (3) Worn time outside the instructed timeframe. Since the wearable sensors recorded from the moment the charger was removed until the moment the charger was plugged in again, there will be time measured in which the participant did not wear the wearable. In addition, patients may have taken off the wearable sensors during daytime when they were fore example taking a shower. In order to only select the data in which the wearable sensors were actually worn, the standard deviation of the acceleration data was calculated per minute block. This was calculated for data in the x direction, which was parallel to the lower arm for the wrist sensors and which was parallel to the whole body for the chest sensor. A threshold value was empirically determined based on the standard deviation of non-worn wearable recordings. As a result, when the standard deviation per minute block was <0.002 g, and when the standard deviation of the preceding and following minute were <0.002 g as well, the minute was not included in the minutes of collected data. Minutes of collected data were averaged over the three sensors, over days, and over participants.

For the ESM, the outcomes are the percentages of completed questionnaires and the percentages of completed questionnaires for which sensor data from all three sensors was available as well. To evaluate the burden of each continuous questionnaire, we included a question on ‘how disturbing’ the corresponding questionnaire was.

For the combination of ESM and sensor data, we evaluated the results of the cross-validation using the area under the curve of the Receiver-Operator-Characteristic.

### Reporting summary

Further information on research design is available in the Nature Research Reporting Summary linked to this article.

## Supplementary information

Supplementary materials

Reporting Summary

## Data Availability

The data that support the findings of this study are available from the corresponding author upon reasonable request.

## Code availability

The scripts for analysis are available from the corresponding author upon reasonable request.
