# Supplementary material for: Monitoring Parkinson’s disease symptoms during daily life: a feasibility study
Source: NPJ Parkinsons Dis. 2019 Sep 30;5:21. doi: 10.1038/s41531-019-0093-5 (PMC6768992; doi:10.1038/s41531-019-0093-5)
Supplement: Supplementary file 1 — Supplementary materials [file 41531_2019_93_MOESM1_ESM.pdf]

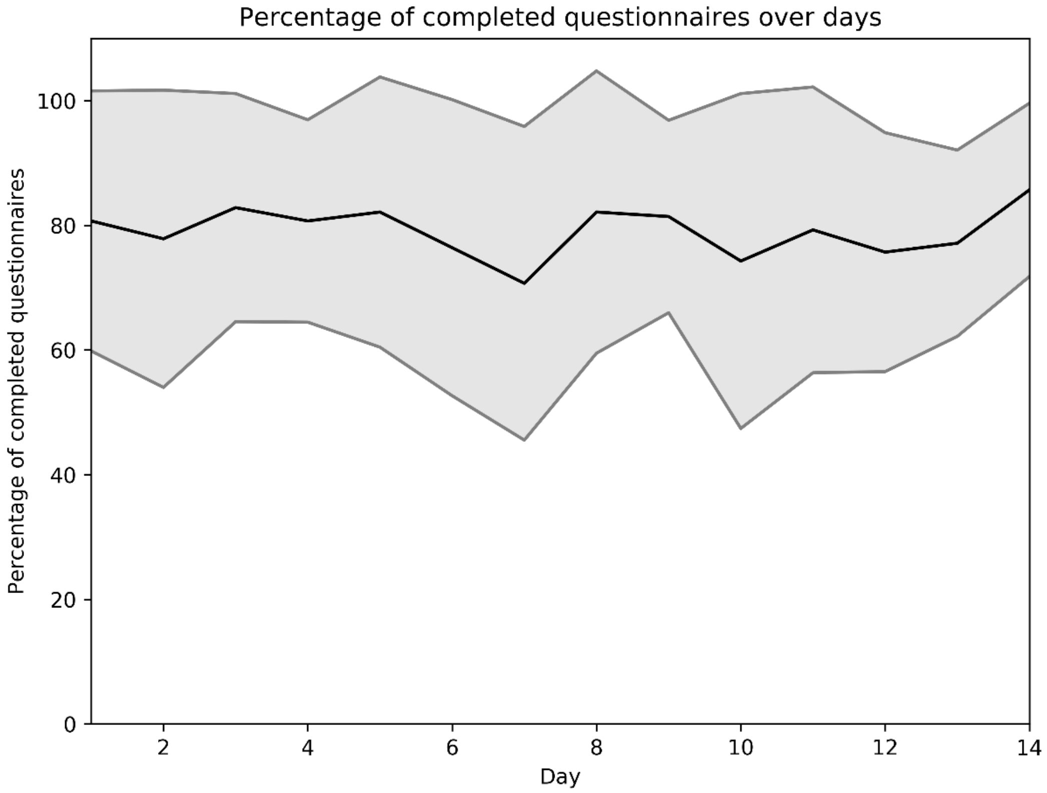

Supplementary Figure 1. Percentage of completed questionnaires over days. Light grey area is showing the standard deviation. A repeated measures ANOVA determined that completion rates did not differ significantly between days ($F(13,247) = 0.97, p = 0.49$).

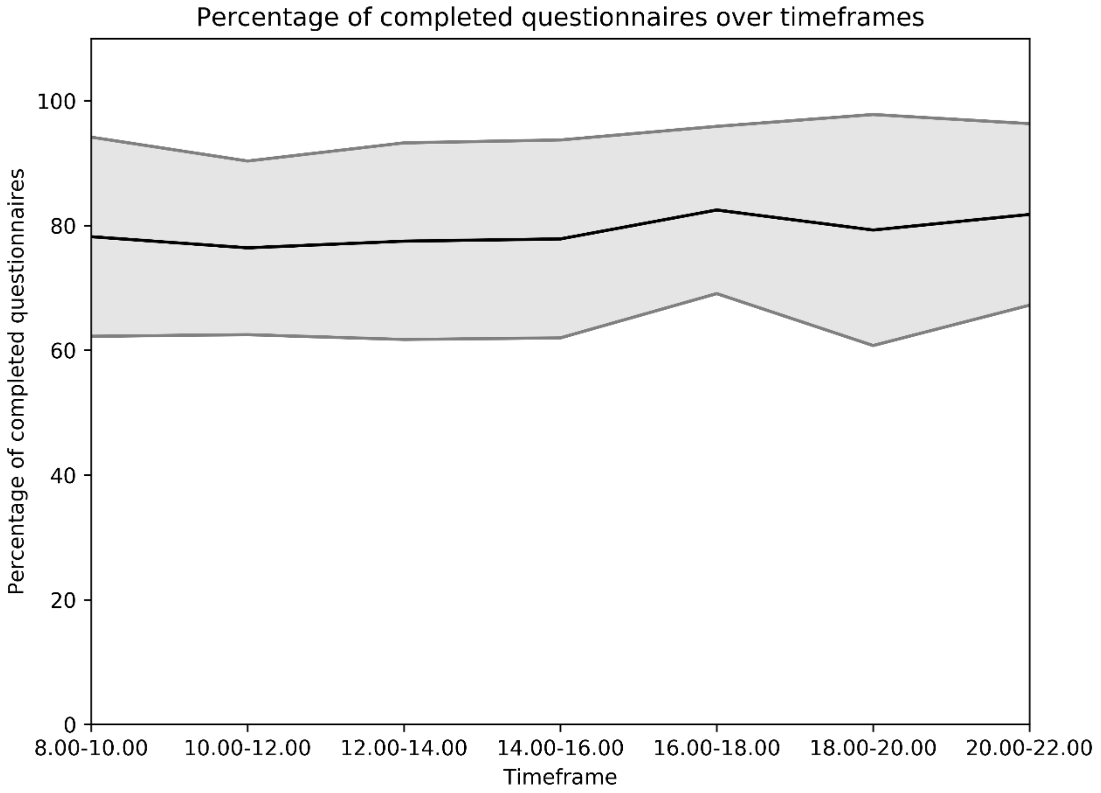

Supplementary Figure 2. Percentage of completed questionnaires over timeframes. Light grey area is showing the standard deviation. A repeated measures ANOVA determined that completion rates did not differ significantly between timeframes ($F(6,114) = 1.20, p = 0.31$).

Continuous questionnaire:

- I feel well
- I feel down
- I feel fearful
- I feel stressed
- I feel sleepy
- I am tired
- I am cheerful
- I am relaxed
- I can concentrate well
- I experience hallucinations
- I am at (home, work, travelling, at family/friends place, in public)
- I am (alone, with family, with my partner, with colleagues, with friends)
- I am doing (work, resting, household/odd jobs, sports, something else)
- I can do this without hinder
- I am comfortable walking/standing
- I can sit or stand still easily
- I can speak well
- I can walk well
- I experience tremor
- I am moving slow
- I experience stiffness
- I experience muscles tension
- I am moving uncontrollably
- I feel currently (OFF, OFF → ON, ON, ON → OFF)
- I took Parkinson medication since the last beep (yes, no, I don't recall)
- I found this beep disturbing

Morning questionnaire

- I slept well
- I woke up often last night
- I feel rested
- It was physically difficult to get up
- It was mentally difficult to get up

Evening questionnaire

- I had long OFF periods today
- I had many OFF periods today
- Walking went well today
- (un)dressing went well today
- Eating and drinking went well today
- Personal care went well today
- Household activities went well today
- I was tired today

Supplementary Figure 3. Experience Sampling Method questionnaires.
